# ABCC1 modulates negative feedback control of the hypothalamic-pituitary-adrenal axis in vivo in humans

**DOI:** 10.1016/j.metabol.2021.155118

**Published:** 2022-03

**Authors:** Catriona J. Kyle, Mark Nixon, Natalie Z.M. Homer, Ruth A. Morgan, Ruth Andrew, Roland H. Stimson, Brian R. Walker

**Affiliations:** a BHF Centre for Cardiovascular Science, Queen's Medical Research Institute, University of Edinburgh, UK; b Mass Spectrometry Core, Edinburgh Clinical Research Facility, Queen's Medical Research Institute, University of Edinburgh, UK; c Translational & Clinical Research Institute, Newcastle University, Newcastle upon Tyne, UK

**Keywords:** Glucocorticoids, ABC transporters, HPA axis, Adipose tissue, Skeletal muscle, 11βHSD, 11β-hydroxysteroid dehydrogenase, ABC, ATP-binding cassette transporters;, ABCB1, ABC transporter B1, ABCC1, ABC transporter C1, ATBF, adipose tissue blood flow, FBF, forearm blood flow, GR, glucocorticoid receptor, HPA, hypothalamic pituitary adrenal, MR, mineralocorticoid receptor, RU486, mifepristone, SGBS, Simpson-Golabi-Behmel syndrome, TTR, tracer:tracee ratio

## Abstract

**Background:**

Cortisol and corticosterone both circulate in human plasma and, due to differing export by ATP-binding cassette (ABC) transporters, may exert differential cellular effects. ABCB1 (expressed in brain) exports cortisol not corticosterone while ABCC1 (expressed in adipose and skeletal muscle) exports corticosterone not cortisol. We hypothesised that ABCC1 inhibition increases corticosteroid receptor occupancy by corticosterone but not cortisol in humans.

**Methods:**

A randomised double-blind crossover study was conducted in 14 healthy men comparing placebo and ABCC1 inhibitor probenecid. Blood sampling, including from veins draining adipose and muscle, was undertaken before and after administration of mineralocorticoid receptor antagonist potassium canrenoate and glucocorticoid receptor antagonist mifepristone (RU486).

**Results:**

During placebo, systemic plasma cortisol and corticosterone concentrations increased promptly after canrenoate. Cortisol uptake was detected from adipose but not muscle following canrenoate + RU486. Probenecid significantly increased systemic cortisol concentrations, and tended to increase corticosterone and ACTH concentrations, after combined receptor antagonism but had no effects on net glucocorticoid balance in either adipose or muscle. Using quantitative PCR in brain bank tissue, *ABCC1* expression was 5-fold higher in human pituitary than hypothalamus and hippocampus. *ABCB1* was more highly expressed in hypothalamus compared to pituitary.

**Conclusions:**

Although displacement of corticosterone and/or cortisol from receptors in adipose and skeletal muscle could not be measured with sufficient precision to detect effects of probenecid, ABCC1 inhibition induced a greater incremental activation of the hypothalamic-pituitary-adrenal axis after combined receptor blockade, consistent with ABCC1 exporting corticosterone from the pituitary and adding to the evidence that ABC transporters modulate tissue glucocorticoid sensitivity.

## Highlights

• Cortisol and corticosterone are glucocorticoids which both circulate in human plasma
• They may exert differential cellular effects due to differing export by ABC transporters
• ABCB1 (in brain) exports cortisol not corticosterone while ABCC1 (in adipose) exports corticosterone not cortisol
• Inhibiting ABCC1 induced greater activation of the HPA axis after combined GR and MR receptor blockade
• This adds to the evidence that ABC transporters modulate tissue glucocorticoid sensitivity

## 1 Introduction

Glucocorticoids play a major role in carbohydrate, protein and lipid metabolism and have significant anti-inflammatory and immunological actions [1]. Cortisol is the principal glucocorticoid in humans regulated systemically by the hypothalamic-pituitary-adrenal (HPA) axis and locally, for example, by the 11β-hydroxysteroid dehydrogenase enzymes (11βHSDs) [2,3]. In adipose tissue, glucocorticoids are central to regulation of lipolysis, adipocyte proliferation and fat accumulation [4] while in skeletal muscle, glucocorticoids regulate protein metabolism and insulin sensitivity. Dysregulation of these processes is implicated in the development of obesity and metabolic syndrome [5].

A neglected area of human glucocorticoid biology is the role of corticosterone. Circulating at ~10-fold lower concentrations than cortisol [6,7], corticosterone had been assumed to mimic cortisol in action and effect and to be under similar control by the HPA axis and 11β-HSD metabolism, and so has been largely ignored. However, corticosterone and cortisol concentrations vary differently in humans, for example with corticosterone more acutely responsive to ACTH and being more rapidly cleared from the circulation [8,9]. Moreover, there is evidence that cortisol and corticosterone act differently on target tissues because they are subject to differential transmembrane transport by adenosine triphosphate binding cassette (ABC) transporters. ABCB1 is most highly expressed in brain, adrenals and small intestine and exports cortisol but not corticosterone [6,[10], [11], [12]]. ABCC1 is more widely expressed although primarily in adipose tissue, skeletal muscle and thyroid and conversely exports corticosterone but not cortisol [11,13,14].

In brain, the predominant expression of ABCB1 over ABCC1 at the blood brain barrier suggests a more significant role for corticosterone in hypothalamic negative feedback [6]. This is supported by evidence of increased corticosterone:cortisol ratio in cerebral spinal fluid (CSF) [15] and post-mortem brain specimens [6] compared to ratios in the circulation such that, in contrast with corticosterone contributing just 5–10% of circulating glucocorticoid, corticosterone accounts for ~30% of glucocorticoid levels in the brain.

Conversely, selective ABCC1 rather than ABCB1 expression in skeletal muscle and adipose tissue may minimise the effects of corticosterone in these tissues, reducing glucocorticoid action by up to 10%. Indeed, pharmacological inhibition and genetic deletion of ABCC1 increased corticosterone activation of corticosteroid receptors in SGBS adipocytes (an established model of human adipocytes) and murine adipose tissue [13].

To test the relevance of tissue-specific export of corticosterone to glucocorticoid signalling in humans we previously conducted a study of patients with Addison's disease, in whom corticosterone and cortisol infused to achieve similar circulating concentrations resulted in comparable suppression of ACTH but cortisol induced higher expression of the glucocorticoid-responsive transcript *PER1* in adipose tissue than corticosterone [13]. These findings are consistent with ABCC1-mediated export of corticosterone from adipose tissue cells, but not from cells involved in HPA axis negative feedback, leading to reduced activation of corticosteroid receptors. Further, they support the over-arching hypothesis that excursions in corticosterone in plasma may have a disproportionate effect on the HPA axis and brain via central feedback while excursions in cortisol have disproportionate effects on metabolic tissues.

However, the optimal test of the contribution of ABCC1 and corticosterone to glucocorticoid signalling in humans requires the use of receptor antagonists to block endogenous steroid action rather than pharmacological infusion of steroids. Here, we used this approach to determine whether inhibition of ABCC1 affects the central negative feedback of the HPA axis and occupancy of the corticosteroid receptors in adipose tissue and skeletal muscle in humans.

## 2 Material and methods

To infer occupancy of the glucocorticoid (GR) and mineralocorticoid receptors (MR) by cortisol and corticosterone, we administered the GR antagonist mifepristone (RU486) and the MR antagonist potassium canrenoate, and measured activation of the HPA axis and displacement of bound glucocorticoids from adipose tissue and skeletal muscle. We combined arteriovenous sampling with use of 1,2-[^2^H]_2_-cortisone (D2-cortisone) because this tracer does not bind to corticosteroid receptors [16,17]. Blood flow was calculated in adipose tissue and skeletal muscle to allow rate of appearance calculations, as per previous measures of occupancy of MR by cortisol in the myocardium [18]. ABCC1 inhibition was achieved using the uricosuric agent probenecid which has been used experimentally to inhibit ABCC1 both in vitro and in vivo [13,14].

### 2.1 Sample size

Statistical power was based on data from a study of myocardial glucocorticoid release after MR blockade [18]. The mean change in cortisol concentration from steady state to maximal release was normally distributed at 55.3 nmol/L with a standard deviation of 69.1. Based on this, 14 pairs of subjects were required in order to reject the null hypothesis that this response difference was zero with power of 0.8 and type 1 error probability of 0.05.

### 2.2 Clinical protocol

Approval from the South East Scotland research ethics committee and written informed consent from each participant were obtained.

Healthy male volunteers (*n* = 14) were recruited to a randomised double-blind placebo-controlled crossover study. Inclusion criteria were males aged 18–60 years and BMI 20–30 kg/m^2^. Exclusion criteria were any acute or chronic medical condition, glucocorticoid treatment by any route in last 3 months, abnormal full blood count, liver, kidney and thyroid function and plasma glucose concentrations, any regular medication and alcohol intake >21 units/week.

Participants attended on two occasions at least three weeks apart to allow washout between visits. They took either placebo or probenecid (Arena Pharmaceuticals Ltd., Buckingham, UK) in random order prior to each study visit. Capsules were manufactured by Tayside Pharmaceuticals in sterile conditions. Participants were given written instructions to take 2 × 500 mg capsules twice daily (8 am and 8 pm) for 5 days before each study visit, with the last 2 capsules taken on the morning of the visit. Randomisation was undertaken by Tayside Pharmaceuticals and kept securely in a sealed envelope until all measurements were complete. Probenecid was well tolerated by all participants.

Participants attended the clinical research facility after overnight fast. Body fat was measured by bioimpedance (Body Fat Monitor BF302; OMRON Healthcare (UK) Ltd., Henfield, UK). A standardised breakfast was supplied at *t* = −30 min consisting of 55% carbohydrate, 30% fat and 15% protein totalling 350 kcal. A cannula (20G) was placed anterogradely into one antecubital fossa vein for infusions. Retrograde 20G cannulae were placed as follows: 1) superficial vein on the anterior abdominal wall, using red light guidance, to sample from subcutaneous adipose; 2) deep branch of the median cubital vein in the contralateral antecubital fossa to sample from forearm skeletal muscle; and 3) dorsal vein of one hand with the hand warmed to 60 °C to sample arterialised blood.

**Fig. 1:**
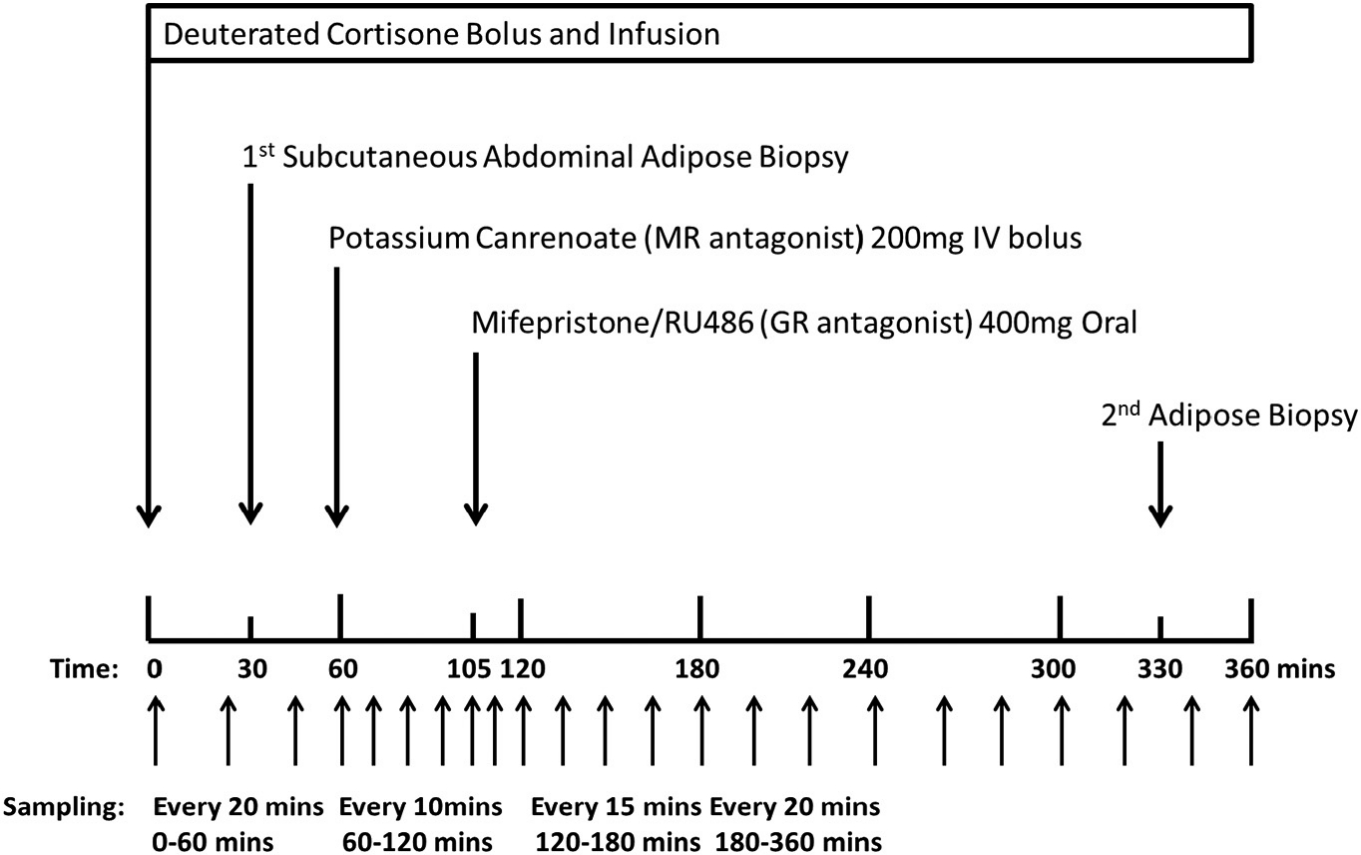
Study protocol.

D2-cortisone was administered at *t* = 0 min in 0.9% saline, as an intravenous bolus of 0.076 mg followed by a continuous infusion of 0.1053 mg/h (Fig. 1). Potassium canrenoate 200 mg (Boehringer Ingelheim, Ingelheim am Rhein, Germany) was administered as an intravenous bolus at *t* = 60 min and RU486 400 mg (Exelgyn, Paris, France) was taken orally with 100 mL of water at *t* = 105 min. Simultaneous arterio-venous samples were obtained at 10–20 min intervals before and after MR and GR antagonist administration. Serum and plasma were stored at −80 °C. Forearm blood flow (FBF) was measured by venous occlusion plethysmography [19] (Hokanson, Bellevue, WA) at hourly intervals as previously described [17]. Adipose tissue blood flow (ATBF) was measured continuously during the study using a Mediscint ɣ-counter probe following a subcutaneous injection of 2 MBq ^133^Xe (IDB Holland) lateral to the umbilicus. At *t* = 30 and 330 min, a needle aspiration biopsy of subcutaneous abdominal adipose tissue was obtained as previously described and stored at −80 °C [20].

### 2.3 Chemical and reagents

/ce:inf>-cortisol, D4-cortisol) and deuterated cortisone (1,2-[^2^H]_2_-cortisone, D2-cortisone) were supplied by Cambridge Isotopes (Tewksbury, MA, USA). Mifepristone (RU486), canrenone, alfaxolone and deuterated canrenone (2,2,4,7,22,22-[^2^H]_6_-canrenone, D6-canrenone) were supplied by Sigma Aldrich (St. Louis, MO, USA). Each of these steroids were weighed and dissolved in methanol (final concentration 1 mg/mL) and stored at −20 °C.

### 2.4 Laboratory analysis

#### 2.4.1 LC-MS/MS analysis

Cortisol, cortisone, D2-cortisone, corticosterone, canrenone and RU486 were extracted from plasma (200 μL) enriched with internal standards (D4-cortisol (50 ng), epi-corticosterone (10 ng), alfaxalone (50 ng) and D6-canrenone (50 ng)) and prepared alongside aqueous calibration curves (200 μL) over a total range 0.001–400 ng. Samples and calibrants were extracted by supported liquid extraction (SLE) and analysed by LC-MS/MS using a Nexera X2 Liquid Chromatography system (Shimadzu, UK) coupled to a QTRAP® 6500+ mass spectrometer (Sciex, Framingham, MA, USA). Briefly, samples and calibrants were diluted 1:1 with HPLC grade water (200 μL), transferred to an SLE 400+ 96-well plate (Biotage, Uppsala, Sweden) and allowed to equilibrate (10 min). The plate was subject to vacuum (5 min). Dichloromethane/propan-2-ol (98:2; 0.9 mL) was applied to each well twice and eluate collected into a 2 mL deep well collection plate (Waters, UK). The eluate was reduced to dryness (OFN, 40 °C) on an SPE Dry™ Dual Sample Concentrator System (Biotage, Uppsala, Sweden). The dried extracts were dissolved in water/methanol (70:30, 70 μL), the plate sealed and shaken (10 min) before injecting (20 μL) directly from the 96-well plate for LC-MS/MS analysis. Chromatographic separation was achieved on an ACE Excel C18 AR column (150 × 2.1 mm, 2 μm; ACT Technologies, Aberdeen, UK). Mass transitions were monitored during selected reaction monitoring (positive electrospray mode; spray voltage 5500 V; source temperature 700 °C and source gases 40:60 psi) as follows: cortisol, *m*/*z* 363.1 → 121.1; corticosterone, *m*/*z* 347.1 → 121.1; D2-cortisone, *m*/*z* 363.1 → 165.1; canrenone, *m*/*z* 341.1 → 107; RU486, *m*/*z* 430.2 → 134; D6-canrenone, *m*/*z* 347 → 107; D4-cortisol, *m*/*z* 366.9 → 121; epi-corticosterone, *m*/*z* 347.1 → 121; alfaxolone, *m*/*z* 333 → 297.

#### 2.4.2 ACTH analysis

Plasma ACTH was quantified by enzyme-linked immunosorbent assay (Biomerica, California, USA).

#### 2.4.3 Quantitative real time PCR measurement in adipose tissue and brain samples

Glucocorticoid sensitive transcripts were quantified in adipose tissue. Human brain tissue from six male subjects consisting of hypothalamus, hippocampus and pituitary was obtained post-mortem (Brain Bank, Centre for Clinical Brain Sciences, University of Edinburgh; Table 1).

**Table 1:** Brain bank samples comprising hypothalamus, hippocampus and pituitary. All samples from male patients.

MRC database number (BBN)	Age (y)
30,916	71
30,841	40
30,208	73
30,178	72
30,169	48
30,147	67

RNA was extracted from brain (750 ng) and adipose (250 ng) and qPCR performed [20]. Primer sequences and probe numbers are summarised in Table 2. Samples were analysed in triplicate, deemed acceptable if the standard deviation of the crossing point was <0.5 cycles. Transcript levels are presented as the ratio of abundance of the gene of interest: mean abundance of control genes. Control genes, *PPIA* and *RNA18S* were used for both tissue types with the addition of *GADPH* for brain tissue samples.

**Table 2:** Primer sequence for qPCR and corresponding probe number for genes of interest from Roche Universal Probe Library (UPL®) for adipose and brain tissue samples.

Gene: name	Primer sequence		UPL probe
*PLIN1*: perilipin	Forward	AGGATGGCAGTCAACAAAGG	42
	Reverse	GCAGCACATTCTCCTGCTC	
*ACAB*: acetyl-CoA carboxylase	Forward	CAGACGCTACAGGTCCCAAC	37
	Reverse	CTGTCCACTCCACTGTCAGG	
*FAS*: Fas cell surface death receptor	Forward	CAGGCACACACGATGGAC	11
	Reverse	CGGAGTGAATCTGGGTTGAT	
*ADIPOQ*: adiponectin	Forward	GGTGAGAAGGGTGAGAAAGGA	85
	Reverse	TTCACCGATGTCTCCCTTAG	
*PNPLA2*: adipose triglyceride lipase	Forward	CTCCACCAACATCCACGAG	89
	Reverse	CCCTGCTTGCACATCTCTC	
*PER1*: period circadian	Forward	CTCTTCCACAGCTCCCTCA	87
	Reverse	CTTTGGATCGGCAGTGGT	
*LPL*: lipoprotein lipase	Forward	ATGTGGCCCGGTTTATCA	25
	Reverse	CTGTATCCCAAGAGATGGACATT	
*PCK1*: phosphoenolpyruvate carboxykinase	Forward	CGAAAGCTCCCCAAGTACAA	20
	Reverse	GCTCTCTACTCGTGCCACATC	
*ABHD5*: abhydrolase domain containing 5	Forward	GGACAAAATGATCTTGCTTGG	66
	Reverse	CCCAAGGCTCCACTAAAATG	
*NR3C1*: nuclear receptor subfamily 3, group C, member 1 (α glucocorticoid receptor)	Forward	TTTTCTTCAAAAGAGCAGTGGA	11
	Reverse	GCATGCTGGGCAGTTTTT	
*NR3C2*: nuclear receptor subfamily 3, group C, member 2 (mineralocorticoid receptor)	Forward	CATCATGAAAGTTTTGCTGCTACT	64
	Reverse	TCTTTGATGTAATTTGTCCTCATTTC	
*SGK1*: serum and glucocorticoid-regulated kinase 1	Forward	GACAGGACTGTGGACTGGTG	24
	Reverse	TTTCAGCTGTGTTTCGGCTA	
*FKBP5*: FK506 binding protein 5	Forward	GGATATACGCCAACATGTTCAA	15
	Reverse	CCATTGCTTTATTGGCCTCT	
*LIPE:* hormone sensitive lipase	Forward	GGAAGTGCTATCGTCTCTGG	SYBR® Green master mix
	Reverse	GGCAGTCAGTGGCATCTC	
*ABCB1:* ATP binding cassette subfamily B member 1	Forward	AAGGCATTTACTTCAAACTTGTCA	18
	Reverse	TGGATTCATCAGCTGCATTTT	
*ABCC1:* ATP binding cassette subfamily C member 1	Forward	GCCTATTACCCCAGCATCG	28
	Reverse	GATGCAGTTGCCCACACA	
*MT1:* metallothionein 1	Taqman Gene expression assay Hs04401199_s1 (Thermo Fisher Scientific)		

*Housekeeping genes*			
*RNA18S:* ribosomal RNA 18s	Forward	CTTCCACAGGAGGCCTACAC	46
	Reverse	CGCAAAATATGCTGGAACTTT	
*PPIA:* peptidylpropyl isomerase A (cyclophilin A)	Forward	ATGCTGGACCCAACACAAAT	48
	Reverse	TCTTTCACTTTGCCAAACACC	
*GAPDH:* glyceraldehyde-3-phosphate dehydrogenase	Forward	AGCCACATCGCTCAGACAC	60
	Reverse	GCCCAATACGACCAAATCC	

### 2.5 Kinetic analysis

ATBF was calculated as previously described [21].

For FBF, calibration of the strain gauge was performed prior to each study visit so that 1% change in length of the gauge was equal to 1% change in limb volume. FBF (mL/100 mL tissue/min) was calculated from the slope of the voltage-time curve from the strain gauge using LabChart Reader (Version 8) software (AD Instruments, Oxford, UK). At least 3 measurements were taken hourly to determine the mean flow rate.

The tracer D2-cortisone was used to allow measurement of flux of cortisol and corticosterone (‘tracees’). The tracer:tracee ratio (Eq. (1)) was used for compartmental modelling calculations and to infer if endogenous tracees were in steady state.

Tracer:tracee ratio (TTR) in arterialised (A) samples

(1) $$\begin{array}{c} \mathrm{TTR}\;\mathrm{Cortisol}=\frac{D2\mathrm{cortisone}\left[A\right]}{\mathrm{Cortisol}\left[A\right]}\\ \mathrm{TTR}\;\mathrm{Corticosterone}=\frac{D2\mathrm{cortisone}\left[A\right]}{\mathrm{Corticosterone}\left[A\right]} \end{array}\;$$

Clearance was calculated for D2-cortisone (Eq. (2)) using arterialised samples and compared to previously published clearance data for cortisol and corticosterone. Steady state (SS) was achieved after 165 min.

Clearance of D2-cortisone

(2) $$\mathrm{Clearance}\;\left(\mathrm{Litres}/\min\right)=\frac{\mathrm{Rate of}\;D2\;\mathrm{cortisone Infusion}\;\left(\mathrm{nmol}/\min\right)}{\mathrm{SS}\;D2\;\mathrm{cortisone Concentration}\;\left[A\right]\;\left(\mathrm{nmol}/L\right)}$$

Rate of appearance (Ra) of cortisol and corticosterone were calculated by dividing the rate of infusion of tracer by the corresponding TTR [17]. Whole body rate of appearance of glucocorticoids were calculated using both steady state equations (Eqs. (3), (4)).

To calculate steady state rate of appearance, tracer and tracee clearance are assumed the same, as these are usually cancelled out. Clearance of D2-cortisone was calculated at 1.3 ± 0.2 L/min, similar to previously reported [17] and clearance of cortisol has been consistently reported at 0.28–0.33 L/min [22,23]. We previously calculated the clearance of deuterated corticosterone (2,2,4,6,6,17α,21,21-[^2^H]_8_-corticosterone) in healthy volunteers during steady state infusions (1.11 ± 0.20 L/min) [24]. Cortisol clearance is approximately 4-fold slower than D2-cortisone and this was corrected using Eq. (3). The clearance rates of corticosterone and D2-cortisone were similar therefore the rate of appearance of corticosterone was calculated without any correction in Eq. (4).

Whole body rate of appearance of cortisol

(3) $$\mathrm{Ra}\;\mathrm{Cortisol}\;\left(\mathrm{nmol}/\min\right)=\frac{\mathrm{Rate}\mathrm{of}\;D2c\mathrm{ortisone}\mathrm{infusion}\;\left(\mathrm{nmol}/\min\right)}{\mathrm{TTR}\;\mathrm{cortisol}*4}$$

Whole body rate of appearance of corticosterone

(4) $$\mathrm{Ra}\;\mathrm{Corticosterone}(\mathrm{nmol}/\min)=\frac{\mathrm{Rate of}\;D2c\mathrm{ortisone}\mathrm{infusion}\;\left(\mathrm{nmol}/\min\right)}{\mathrm{TTR}\;\mathrm{corticosterone}}$$

The rate of appearance of cortisol and corticosterone across tissues (skeletal muscle and adipose tissue) was calculated using arteriovenous differences in TTR while factoring in blood flow (BF) rate through the tissue (Eq. (5)).

Rate of appearance of glucocorticoid across tissue

(5) $$\mathrm{Ra}\;\mathrm{Glucocorticoid}\left(\mathrm{GC}\right)\;\mathrm{across tissue}\;\left(\mathrm{pmol}/100g\;\mathrm{tissue}/\min\right)=\left(\mathrm{BF}\times \left[{\mathrm{GC}}_{\mathrm{Artery}}\right]\times \frac{{\mathrm{TTR}}_{\mathrm{Artery}}}{{\mathrm{TTR}}_{\mathrm{Tissue vein}}}\right)-\mathrm{BF}\times \left[{\mathrm{GC}}_{\mathrm{Artery}}\right]$$

Net balance was calculated for each glucocorticoid across adipose tissue and skeletal muscle to quantify either net release or uptake within the tissue, by calculating the difference in arterial and tissue vein glucocorticoid concentration and multiplying this by tissue blood flow (Eq. (6)).

Net balance of glucocorticoid across tissue

(6) $$\mathrm{Net}\;\mathrm{Balance}\;\left(\mathrm{pmol}/100g\;\mathrm{tissue}/\min\right)=\left[\left[{\mathrm{GC}}_{\mathrm{Tissue vein}}\right]-\left[{\mathrm{GC}}_{\mathrm{Artery}}\right]\right]\times \mathrm{BF}$$

### 2.6 Statistical analysis

All data are mean ± SEM unless otherwise stated. Data were analysed using Graph Pad Prism® (version 6.01) and checked for normality of distribution using Kolmogorov-Smirnov tests. If not normally distributed, data were log transformed prior to analysis. Differences between placebo and probenecid groups were assessed using repeated measures analysis of variance (ANOVA) with post-hoc Bonferroni correction. To account for missing data, average values were calculated for each subject in three time periods before statistical analysis: 1) pre-drug (*t* = 0–60 min); 2) following potassium canrenoate (*t* = 70–105 min); 3) following potassium canrenoate plus RU486 (combined receptor antagonism) (*t* = 110–360 min). Transcript abundances in adipose and brain tissue were compared using repeated measures ANOVA with post-hoc Bonferroni correction. *P* < 0.05 was considered significant.

## 3 Results

### 3.1 Participant characteristics

Participants (*n* = 14) were aged 28.7 ± 3.6 years with body mass index of 24.1 ± 0.7 kg/m^2^ and fat mass 11.2 ± 1.2 kg. Baseline blood pressure was 136.5 ± 1.2 mmHg systolic and 81.1 ± 1.5 mmHg diastolic. Arterialised blood and skeletal muscle data are presented from all 14 participants. Abdominal adipose vein cannulation was unsuccessful on at least 1 visit in 7 subjects and thus adipose tissue data are presented from those 7 participants with data from both visits. Adipose biopsy analysis includes 12 of 14 participants. One participant had low abdominal adiposity so biopsy was not attempted and another had poor yield from one sample and was therefore excluded from analysis.

### 3.2 Systemic measurements

#### 3.2.1 Arterialised glucocorticoid and ACTH concentrations

**Fig. 2:**
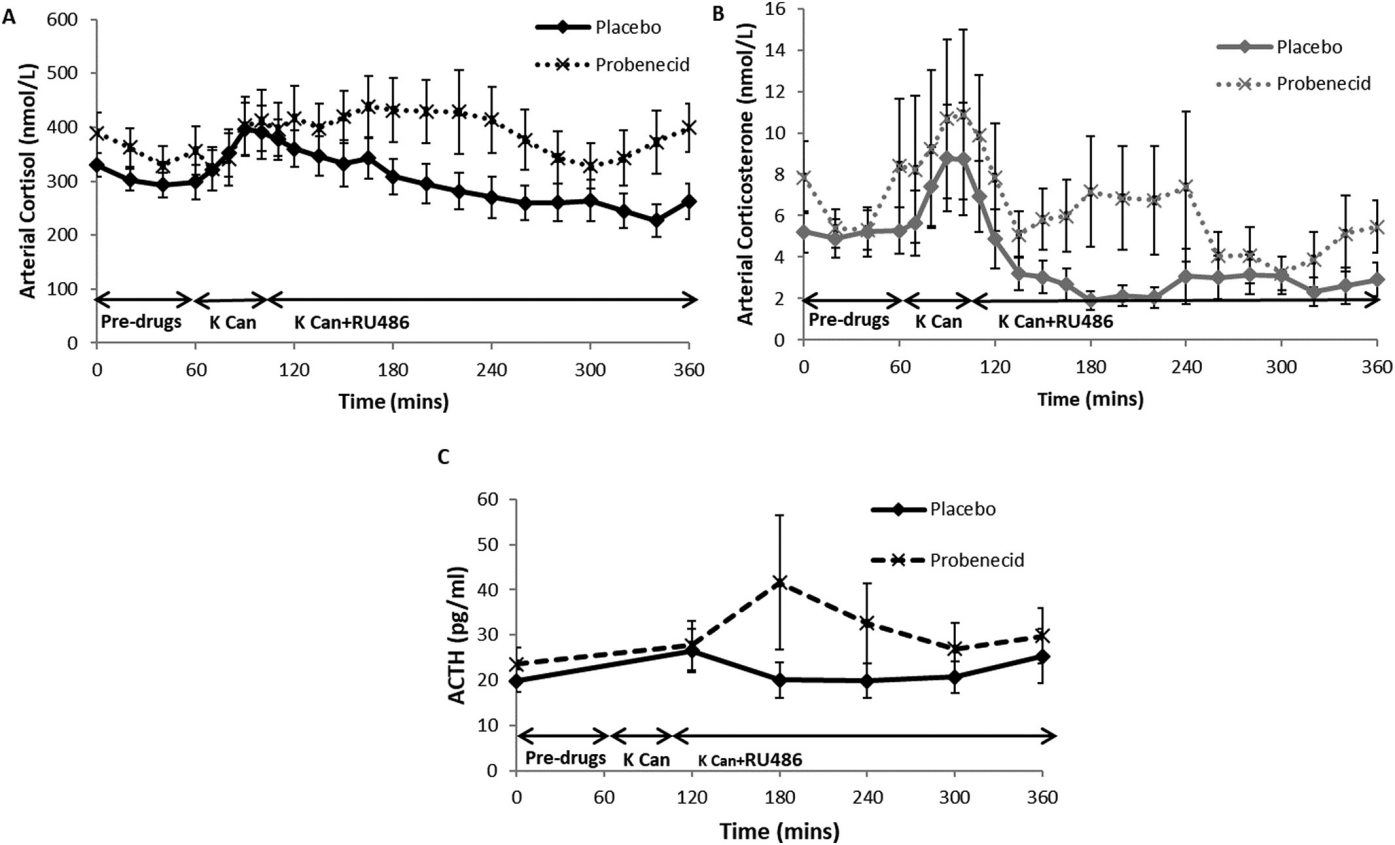
Effect of ABCC1 inhibition on whole body cortisol, corticosterone and ACTH concentrations during MR +/− GR antagonism. Data are mean ± SEM for plasma cortisol (A) and corticosterone (B) concentrations in arterialised samples at time points 0–360 min during placebo (unbroken lines) and probenecid phases (dashed lines) (*n* = 14). Potassium canrenoate (K Can) and mifepristone (RU486) increased cortisol concentrations (*p* < 0.001) and probenecid significantly increased cortisol compared to placebo (p = 0.05). K Can and RU486 increased corticosterone concentrations (*p* < 0.001) while probenecid tended to increase corticosterone (p = 0.08). (C) Plasma ACTH concentrations in placebo (black unbroken line) and probenecid phases (black dashed line) (*n* = 14). Probenecid tended to increase ACTH (*p* = 0.05).

During the placebo phase, both cortisol and corticosterone concentrations increased after potassium canrenoate administration and fell steadily thereafter, including after the addition of RU486 (Fig. 2A/B). There was no difference in fasting cortisol and corticosterone between placebo and probenecid phases. With ABCC1 inhibition in the probenecid phase, there were higher cortisol levels (*p* = 0.01) after RU486 administration. Corticosterone showed a similar although non-significant trend (*p* = 0.08).

In the placebo phase, ACTH did not change with time (Fig. 2C). However, ABCC1 inhibition tended to increase ACTH following potassium canrenoate/RU486 (*p* = 0.06).

### 3.3 Adipose tissue

#### 3.3.1 Adipose tissue blood flow

**Fig. 3:**
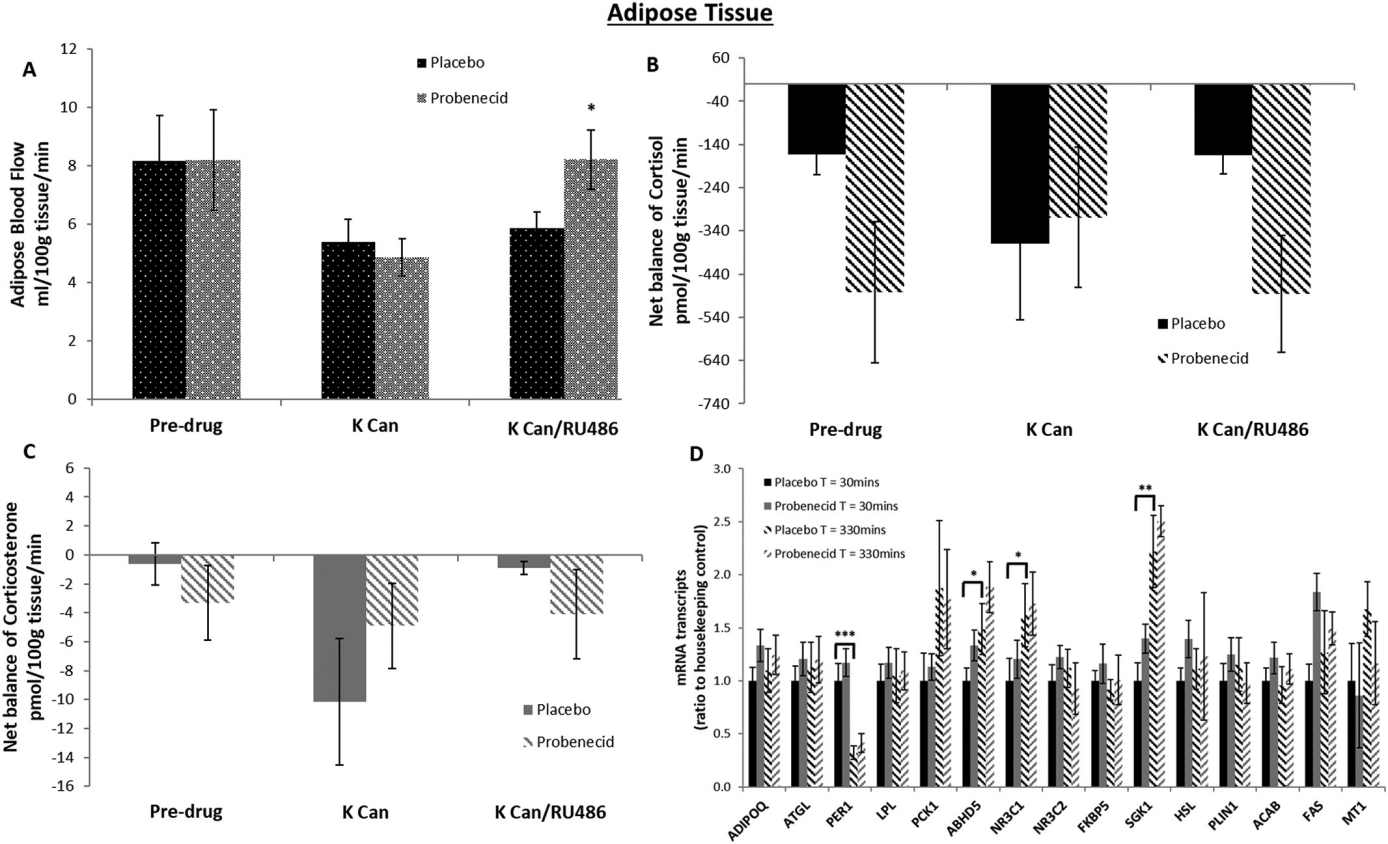
Effect of ABCC1 inhibition on net uptake of cortisol or corticosterone and blood flow during MR +/− GR antagonism and mRNA transcripts in adipose tissue. Data are mean ± SEM. Adipose blood flow (A) in placebo (black spotted fill) and probenecid (grey spotted fill) phases. Probenecid increased blood flow only after combined receptor antagonism (*p < 0.05). Net balance of cortisol (black) and corticosterone (grey) in adipose tissue (*n* = 7) (B and C) comparing placebo (solid fill) and probenecid (striped fill) phases. There was significant uptake of cortisol but not corticosterone throughout both phases (p < 0.02 vs zero). Net balance of cortisol and corticosterone was unaltered by probenecid, canrenoate or RU486. Subcutaneous adipose tissue biopsies at t = 30 (solid fill) and t = 330 (striped fill) minutes in the placebo (black) and probenecid (grey) phases (D). mRNA transcript levels of glucocorticoid responsive genes adiponectin (*ADIPOQ*), adipose triglyceride lipase (*ATGL*), period circadian clock 1 (*PER1*), lipoprotein lipase (*LPL*), phosphoenolpyruvate carboxykinase (*PCK1*), abhydrolase domain containing protein 5 (*ABHD5*), glucocorticoid receptor (*NR3C1*), mineralocorticoid receptor (*NR3C2*), FK506 binding protein 5 (*FKBP5*), serum and glucocorticoid-regulated kinase (*SGK1*), hormone sensitive lipase (*HSL*), perilipin (PLIN1), Acetyl-CoA carboxylase (ACAB), Fas cell surface death receptor (FAS) and metalloproteinase 1 (MT1) (*n* = 12). All samples measured in triplicate and corrected for housekeeping genes *RNA18s* and *PPIA* which were not affected by treatment. *PER1* transcripts reduced significantly (***p < 0.001) between T = 30 and T = 330 min while *ABHD5*, *NR3C1* (both *p < 0.05) and *SGK1* (**p < 0.01) transcript levels were significantly increased. Probenecid did not have any significant effect.

Mean blood flow at baseline (pre-drugs) was similar between placebo and probenecid phases (Fig. 3A). ABCC1 inhibition via probenecid increased blood flow only after combined receptor (potassium canrenoate and RU486) antagonism (*p* = 0.03).

#### 3.3.2 Net balance of glucocorticoids in adipose

There was detectable uptake of cortisol (p = 0.01) but not corticosterone in adipose tissue (Fig. 3B and C). Neither probenecid, potassium canrenoate nor RU486 altered net balance of cortisol or corticosterone.

#### 3.3.3 Glucocorticoid-responsive gene expression in subcutaneous adipose tissue

*PER1* mRNA levels fell significantly from *T* = 30 to 330 min (Fig. 3D). Gene transcripts for *ABHD5*, *NR3C1* (encoding GR) and *SGK1* were significantly higher at *T* = 330 min from baseline. Other glucocorticoid responsive gene transcripts including *ADIPOQ*, *ATGL*, *LPL*, *PEPCK*, *MR*, *FKBP5*, *MT1*, *PLIN1*, *ACAB*, *FAS* and *HSL* were unchanged over the time period. ABCC1 inhibition did not alter any measured mRNA levels.

### 3.4 Skeletal muscle

#### 3.4.1 Skeletal muscle blood flow

**Fig. 4:**
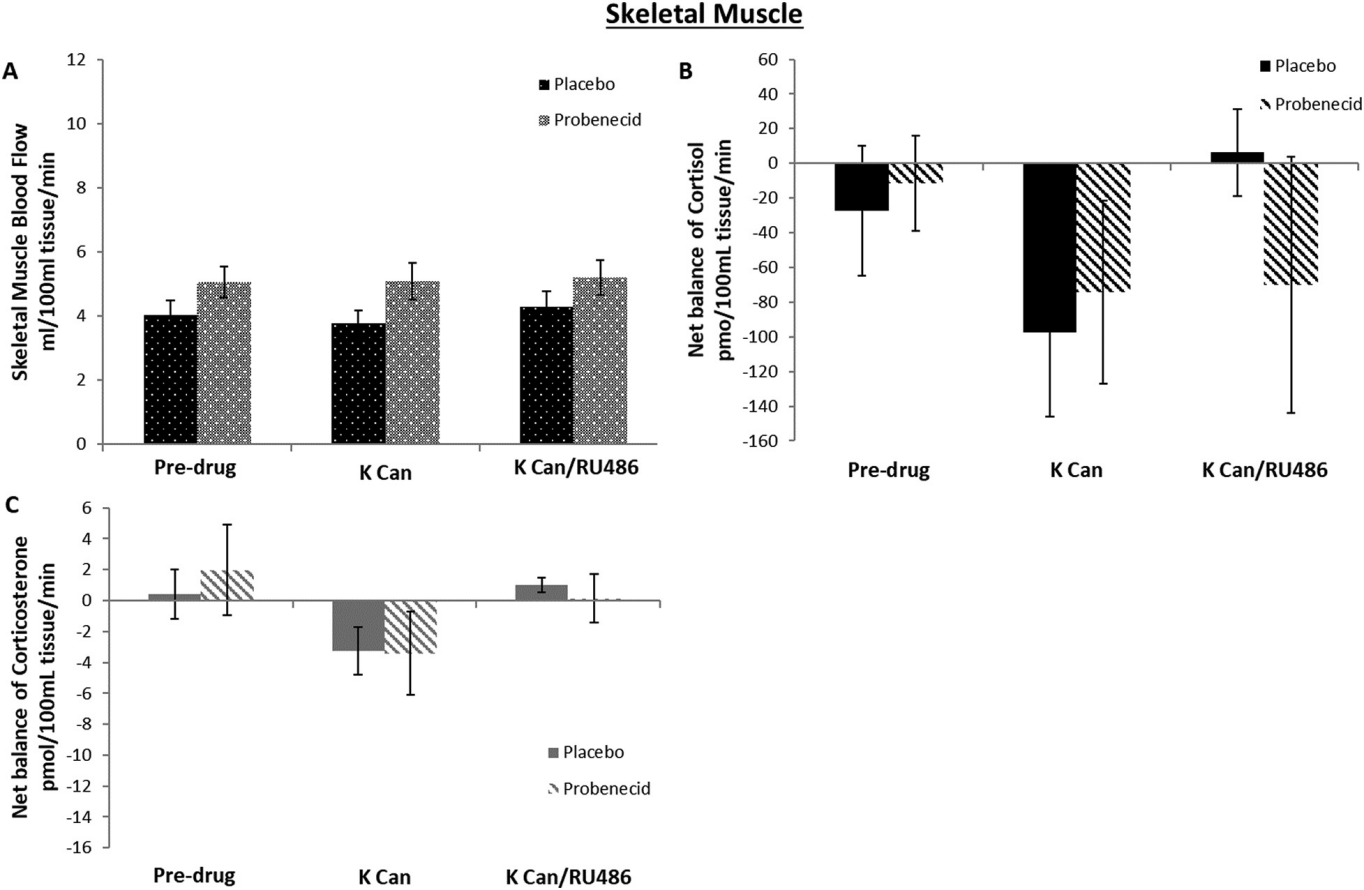
Effect of ABCC1 inhibition on net uptake of cortisol or corticosterone and blood flow during MR +/− GR antagonism in skeletal muscle. Data are mean ± SEM. Skeletal muscle blood flow (B) in placebo (black spotted fill) and probenecid (grey spotted fill) phases. Probenecid increased blood flow throughout (p = 0.03). Net balance of cortisol (black) and corticosterone (grey) in skeletal muscle (n = 14) (B and C) comparing placebo (solid fill) and probenecid (striped fill) phases. There was significant uptake of cortisol but not corticosterone throughout both phases (*p* < 0.02 vs zero). Net balance of cortisol and corticosterone was unaltered by probenecid, canrenoate or RU486.

In the placebo phase, skeletal muscle blood flow did not change with time (Fig. 4A). ABCC1 inhibition increased blood flow compared with placebo throughout the study period (p = 0.03).

#### 3.4.2 Net balance of glucocorticoids in skeletal muscle

There was no significant detectable uptake or release of cortisol or corticosterone across skeletal muscle. Neither canrenoate nor RU486 altered net balance and there was no effect of ABCC1 inhibition with probenecid (Fig. 4B, C).

### 3.5 Rate of appearance of glucocorticoids in systemic circulation, adipose tissue and skeletal muscle

**Fig. 5:**
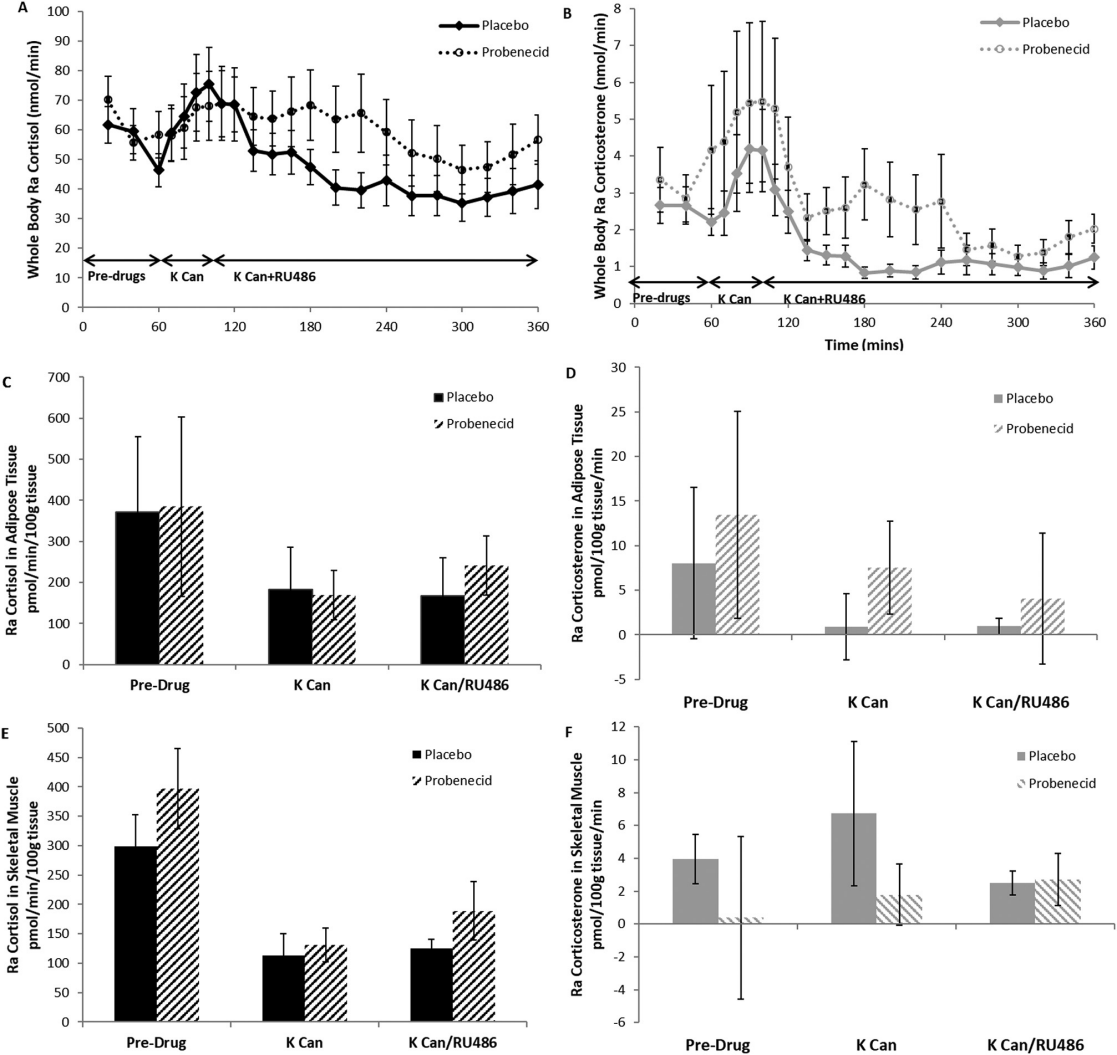
Rate of appearance of cortisol and corticosterone in whole body, adipose tissue and skeletal muscle. Data are mean ± SEM. Whole body rate of appearance (Ra) of cortisol (A) and corticosterone (B) (both n = 14) were compared in placebo (unbroken line) and probenecid (dashed line) phases at time points 0–360 min. Potassium canrenoate (K Can) increased Ra cortisol and corticosterone during the placebo phase (both p < 0.001). Probenecid increased Ra cortisol but not corticosterone following RU486. There was detectable cortisol (C) but not corticosterone (D) generation across adipose tissue (both *n* = 7) but only in the pre-drug period (p < 0.05 vs zero). Probenecid did not alter Ra cortisol or Ra corticosterone across adipose. There was detectable Ra cortisol (E) and Ra corticosterone (F) across skeletal muscle (both n = 14) throughout the study period which was unaltered by probenecid.

Calculated rate of appearance of glucocorticoids in whole body, adipose and skeletal muscle were similar to the data shown in arterialised blood (whole body) and net balance within adipose and skeletal muscle. These are summarised in Fig. 5.

### 3.6 Drug concentrations

**Fig. 6:**
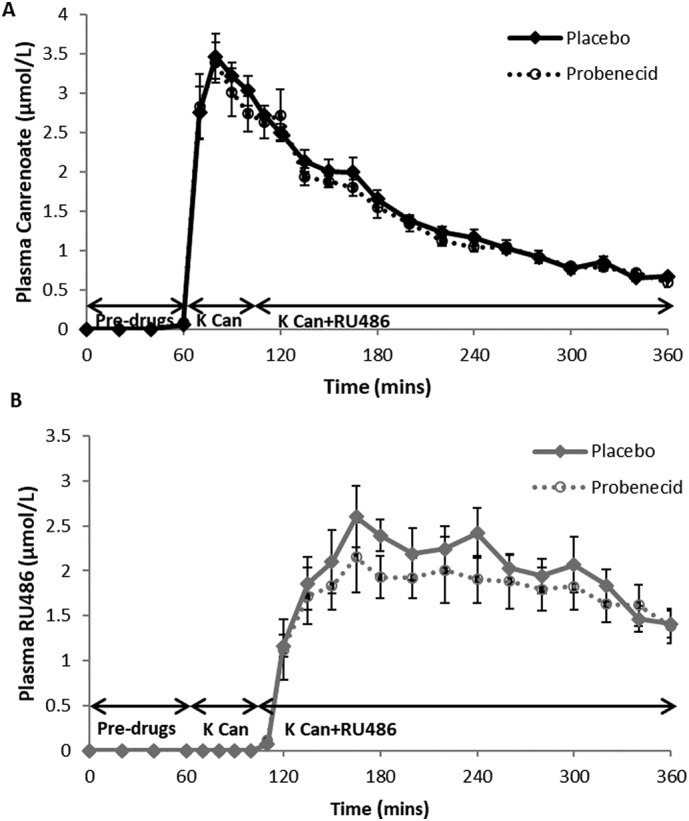
Plasma drug concentrations. Data are mean ± SEM for plasma arterialised concentrations of canrenoate (A) and RU486 (B) during placebo (unbroken line) and probenecid (dashed line) phases (both n = 14). There was no difference in either canrenoate or RU486 concentration between placebo and probenecid phases.

Plasma concentrations of potassium canrenoate and RU486 did not differ during the placebo and probenecid phases (Fig. 6).

### 3.7 ABC transporter expression in post-mortem human brain tissue

**Fig. 7:**
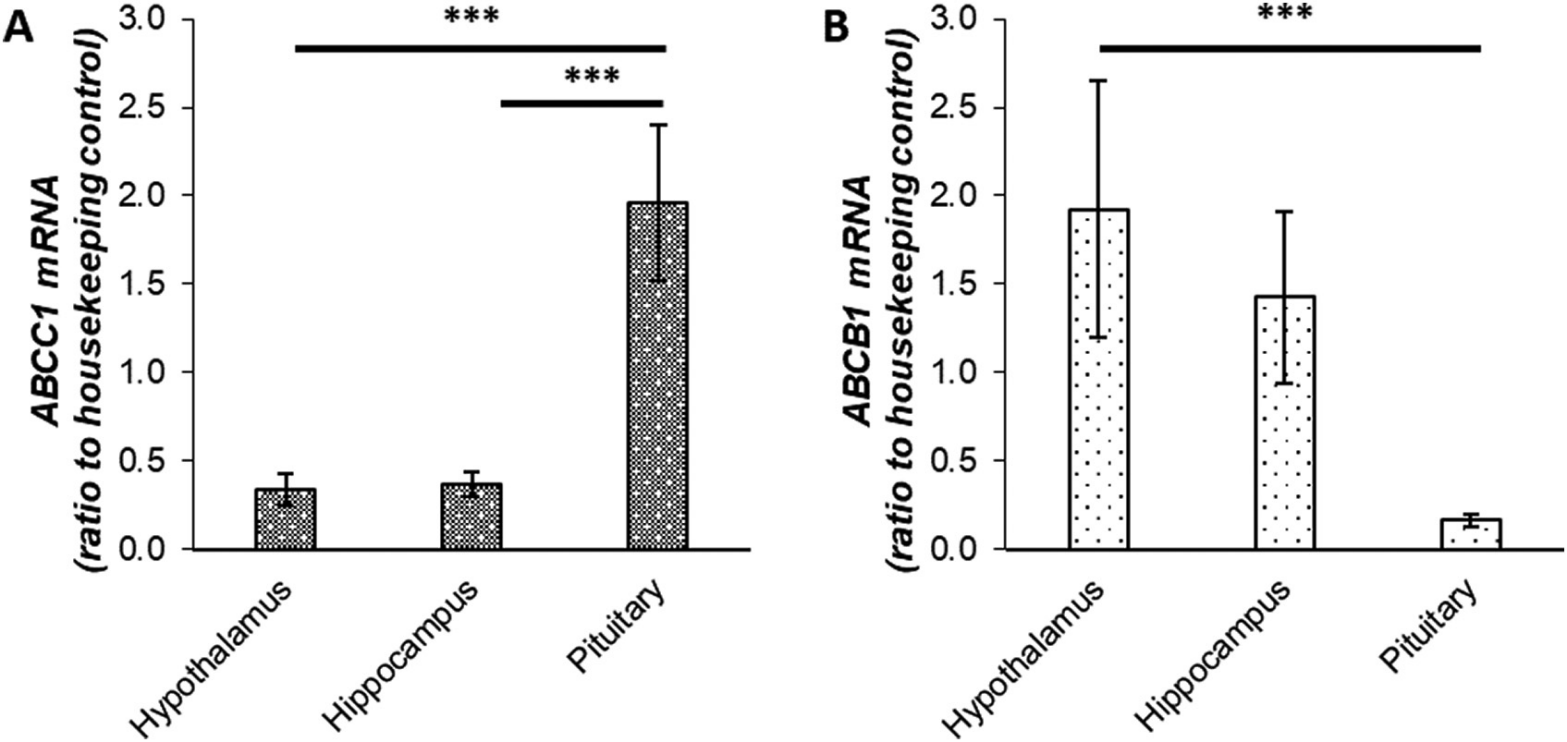
ABC transporter expression patterns in post-mortem brain samples. mRNA transcript levels of *ABCC1* (A) and *ABCB1* (B) in human brain tissue (*n* = 6). All samples measured in triplicate and corrected for housekeeping genes *GADPH*, *RNA18s* and *PPIA* which were not affected by treatment. *ABCC1* transcripts were more highly expressed in pituitary compared to hypothalamus and hippocampus (***p = 0.001). *ABCB1* transcripts were more highly expressed in hypothalamus compared to pituitary (***p = 0.001).

ABCB1 mRNA transcripts were more abundant in the hypothalamus compared to pituitary. In contrast, ABCC1 transcript levels were more abundant in pituitary compared to both hypothalamus and hippocampus (Fig. 7).

## 4 Discussion

**Fig. 8:**
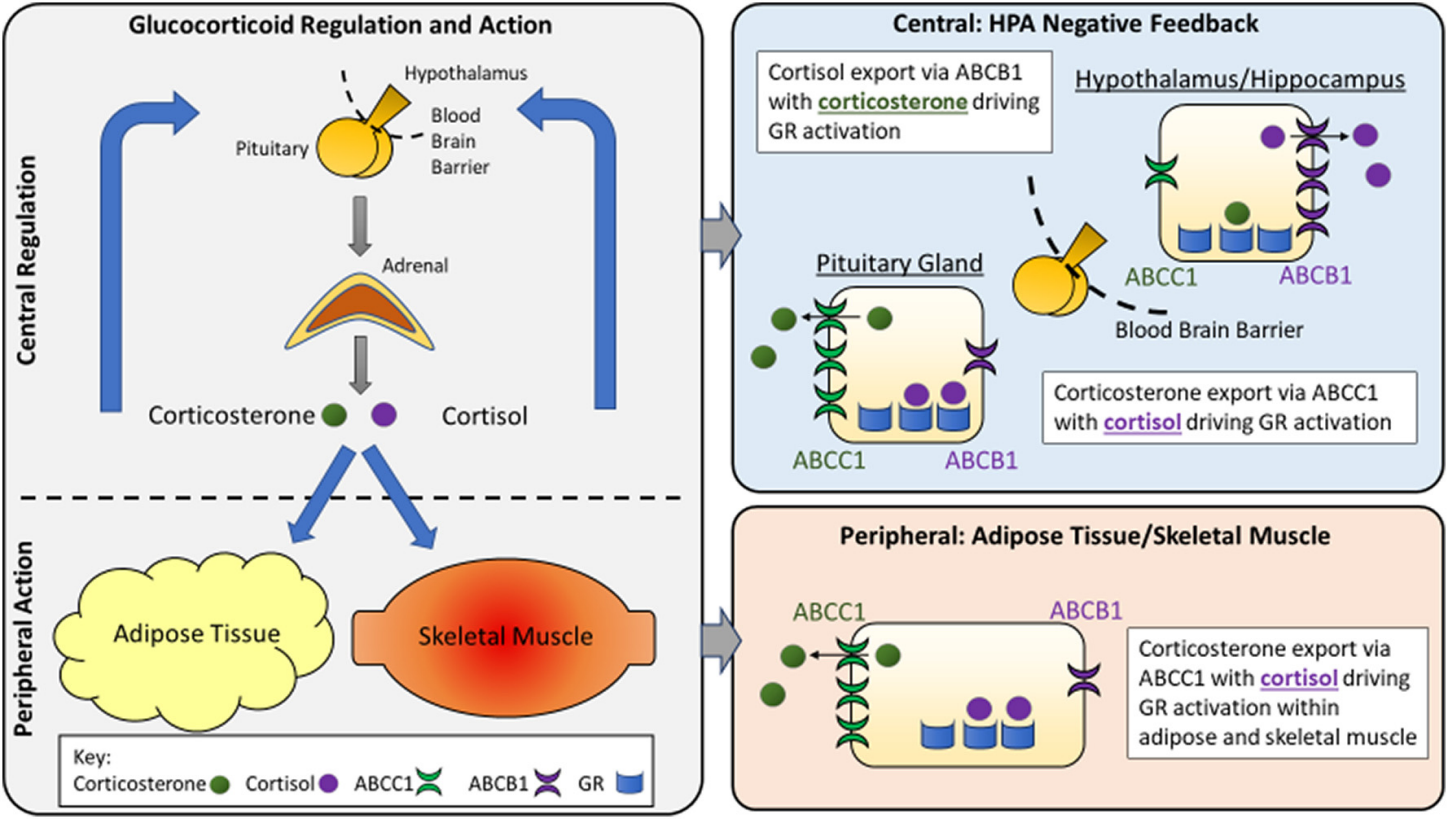
Glucocorticoid regulation and action in central and peripheral tissues. The hypothalamic-pituitary-adrenal (HPA) axis is modulated centrally via negative feedback control within the pituitary and hypothalamus. Peripherally, actions are mediated via the glucocorticoid receptor (GR) within target tissues. We highlight the distinct actions of cortisol and corticosterone within this system. Peripherally, corticosterone is exported via ABCC1 leaving cortisol to drive GR activation within adipose tissue and skeletal muscle. Centrally, in the pituitary (outwith the blood brain barrier), corticosterone is exported via ABCC1 and cortisol drives GR activation. Conversely, within the hypothalamus and hippocampus, ABCB1 predominates, exporting cortisol and leaving corticosterone to drive GR activation.

These data show that ABCC1 inhibition potentiates the activation of the HPA axis which follows combined receptor blockade in humans. We attribute this to preferential expression of ABCC1 over ABCB1 in the human pituitary. We infer that the contribution of corticosterone to pituitary-dependent negative feedback control of the HPA axis is normally constrained by export via ABCC1, so that cortisol may predominate in pituitary-mediated negative feedback whereas corticosterone may have a disproportionately important role in HPA feedback at the hypothalamus (Fig. 8).

This study is the first, to our knowledge, to measure the direct effects of ABCC1 inhibition on glucocorticoid flux in vivo in humans. We hypothesised that inhibition of ABCC1 would have no effect on central negative feedback of the HPA axis given the relative lack of ABCC1 at the blood brain barrier [6,15,25]. In fact, our results showed the opposite with greater activation of the HPA axis by MR and GR antagonism after ABCC1 inhibition. This suggests ABCC1 does play an important role in negative feedback and this effect appears to be centrally mediated with probenecid tending to increase ACTH concentrations, as opposed to a direct effect on the adrenal or any alteration in peripheral clearance of glucocorticoids which would lower ACTH.

To explain this effect, we explored the expression of ABC transporters within different areas of the brain. The significance of ABCB1 in glucocorticoid transport at the blood brain barrier was highlighted when ABCB1 was demonstrated to limit access of cortisol to the brain, suggesting corticosterone played a disproportionately significant role in negative feedback [6]. The corticosterone: cortisol ratio was significantly higher in post-mortem brain samples compared to plasma however specific areas within the brain were not examined [6]. It should be noted that there is limited ABCC1 expression at the blood brain barrier which has been shown to have effects on drug efflux although expression is relatively lower than ABCB1 [[26], [27], [28]]. Analysis of mRNA levels of ABCB1 and ABCC1 in post-mortem brain samples supported our findings and suggest negative feedback within the pituitary is influenced by ABCC1. This interpretation might be considered to be at odds with our previous results showing that cortisol and corticosterone are equally potent at suppressing ACTH during infusion in patients with Addison's disease [13]. However, the relative importance of hypothalamic and pituitary responses may differ in baseline circumstances tested by receptor antagonism versus suppression tested by steroid infusion. Further research is needed both to dissect the level at which ABCC1 influences the HPA axis in different conditions and to determine the role of ABCB1 in HPA axis feedback; however, there is currently no readily available ABCB1 inhibitor to utilise in human studies.

Even though this study benefits from a double-blind crossover study design which mitigates the inter-individual variability in hormone measurements and relatively diverse participant age and BMI, we were unable to measure displacement of corticosterone and/or cortisol from receptors in adipose and skeletal muscle with sufficient precision to detect effects of ABCC1 inhibition. We expected to demonstrate release of glucocorticoids from adipose tissue and skeletal muscle in response to receptor antagonists but observed no change in net balance within either tissue. Release of corticosterone or cortisol was also not observed when we corrected using a tracer, D2-cortisone, in an attempt to enhance precision. The observed steroid uptake most likely reflects the changes in plasma glucocorticoids and gradient into tissues following overnight low plasma glucocorticoid levels. Our adipose tissue data was limited (*n* = 7) due to technical difficulties and was therefore underpowered so may have missed any small corticosteroid release. Our skeletal muscle data, however, was fully powered and displacement was still not demonstrated.

Displacement of glucocorticoids across tissue can be measured, for example, in heart [18] but the increments were very small in that study and it may be difficult to demonstrate in other tissues. Detecting displacement from corticosteroid receptors requires those displaced glucocorticoids to exit the cell and enter the local circulation. It is, of course, possible that there has been displacement from GR and MR but these glucocorticoids remained and were metabolised within the tissue. It is also feasible that the enhanced central effect on the HPA axis stimulating adrenal release of cortisol and corticosterone may have masked any effect of displacement due to the change in circulating concentrations. These additional effects are largely controlled for within the crossover study design.

Data from subcutaneous adipose tissue biopsies provided evidence that corticosteroid receptor blockade was successful but did not provide evidence supporting a role for ABCC1 in modifying receptor occupancy. Expression of *GR* levels increased 1.5-fold following GR and MR antagonism, consistent with its autoregulation in adipose tissue [20]. The glucocorticoid sensitive gene *PER1* fell significantly with time in both phases following receptor antagonism, consistent with its acute induction by glucocorticoids [29]. While this may be interpreted as successful inhibition of GR, given the circadian nature of *PER1* expression, we would need to have had placebo controls for canrenoate and RU486 administration to confirm this; unfortunately, this was considered impractical due to the invasive nature of the protocol. The rise in *ABHD5* and *SGK1* transcript levels following receptor antagonism were unexpected as both are glucocorticoid sensitive, the former a co-factor of key lipase *ATGL* and the latter an early transcriptional glucocorticoid target activated by insulin [20,30,31]. One possible confounding factor was the breakfast provided for participants before the study. This would suppress lipolysis initially but when the second biopsy was taken, lipolysis was likely be increased with ongoing fasting. Although *SGK1* is regulated by glucocorticoids, both acute and chronic inflammation induce expression [32]. Both biopsies were taken from the same area and inflammation from the initial biopsy may have influenced expression in the subsequent sample. Again, these genes may be affected by a circadian rhythm but the expected effect would have been a significant fall with time [33,34]. Probenecid had no effect on adipose transcript expression at baseline or following receptor blockade, although there was a trend for higher expression in the probenecid phase for all genes tested except *MTI* which could indicate increased action of corticosterone within adipose tissue.

The study was designed to identify differences in glucocorticoid release after first MR then GR receptor blockade. We did show canrenoate plasma levels peak just after 60 min compared to RU486 at 150 min which supports our study design but we cannot be certain their effects were maximal at these times.

We must consider whether effects of probenecid might not be mediated by the effect of ABCC1 on corticosteroid transport. Increases in blood flow with probenecid were unexpected and may be explained by its potent agonist effect on transient receptor vanilloid 2 (TRPV2). This plays a significant role in cardiac function and probenecid has potential positive inotropic effects. We did see evidence of increased skeletal muscle blood flow with probenecid however any change in blood flow was accounted for in the rate of appearance calculations and are unlikely to have influenced our results. Probenecid also increased adipose blood flow but not from baseline and only after combined receptor antagonism. This suggests a glucocorticoid-mediated mechanism rather than a direct effect on cardiac output. Probenecid also inhibits a number of cell membrane transporters, including the organic anion transporter which export uric acid [35,36]. However, there was no activation of the HPA axis by probenecid in the absence of corticosteroid receptor blockade making a non-specific mechanism unlikely. Moreover, probenecid does not inhibit ABCB1 [14,28,37]. It was reassuring that systemic concentrations of potassium canrenoate and RU486 were unchanged in the placebo and probenecid phases.

In conclusion, these data represent the first evidence of ABCC1 regulating pituitary-driven HPA negative feedback in vivo in humans. While we were unable to detect effects in peripheral tissues, our data further highlight the potential for discrete roles of cortisol and corticosterone in humans.

## CRediT authorship contribution statement

**Catriona J. Kyle:** Methodology, Investigation, Formal analysis, Writing – original draft, Visualization. **Mark Nixon:** Methodology, Writing – review & editing, Visualization. **Natalie Z. Homer:** Methodology, Resources, Validation, Formal analysis, Writing – review & editing. **Ruth A. Morgan:** Formal analysis, Resources, Writing – review & editing. **Ruth Andrew:** Methodology, Validation, Formal analysis, Writing – review & editing. **Roland H. Stimson:** Methodology, Formal analysis, Visualization, Supervision, Funding acquisition, Writing – review & editing. **Brian R. Walker:** Conceptualization, Methodology, Resources, Formal analysis, Visualization, Supervision, Funding acquisition, Writing – review & editing.

## Declaration of competing interest

The authors declare no conflict of interest that is relevant to the subject matter or materials included in this work.
